# Laboratory Biomarkers for Diagnosis and Prognosis in COVID-19

**DOI:** 10.3389/fimmu.2022.857573

**Published:** 2022-04-27

**Authors:** Denise Battaglini, Miquéias Lopes-Pacheco, Hugo C. Castro-Faria-Neto, Paolo Pelosi, Patricia R. M. Rocco

**Affiliations:** ^1^ Anesthesia and Intensive Care, San Martino Policlinico Hospital, Instituto di Ricovero e Cura a Carattere Scientifico (IRCCS) for Oncology and Neuroscience, Genoa, Italy; ^2^ Department of Surgical Science and Integrated Diagnostics (DISC), University of Genoa, Genoa, Italy; ^3^ Department of Medicine, University of Barcelona, Barcelona, Spain; ^4^ Laboratory of Pulmonary Investigation, Carlos Chagas Filho Biophysics Institute, Federal University of Rio de Janeiro, Rio de Janeiro, Brazil; ^5^ Laboratory of Immunopharmacology, Oswaldo Cruz Institute - Fiocruz, Rio de Janeiro, Brazil; ^6^ COVID-19 Virus Network from Brazilian Council for Scientific and Technological Development, Brasília, Brazil; ^7^ COVID-19 Virus Network from Foundation Carlos Chagas Filho Research Support of the State of Rio de Janeiro, Rio de Janeiro, Brazil

**Keywords:** biomarkers, COVID-19, inflammation, metabolomics, proteomics

## Abstract

Severe acute respiratory syndrome-coronavirus 2 (SARS-CoV-2) causes a wide spectrum of clinical manifestations, with progression to multiorgan failure in the most severe cases. Several biomarkers can be altered in coronavirus disease 2019 (COVID-19), and they can be associated with diagnosis, prognosis, and outcomes. The most used biomarkers in COVID-19 include several proinflammatory cytokines, neuron-specific enolase (NSE), lactate dehydrogenase (LDH), aspartate transaminase (AST), neutrophil count, neutrophils-to-lymphocytes ratio, troponins, creatine kinase (MB), myoglobin, D-dimer, brain natriuretic peptide (BNP), and its N-terminal pro-hormone (NT-proBNP). Some of these biomarkers can be readily used to predict disease severity, hospitalization, intensive care unit (ICU) admission, and mortality, while others, such as metabolomic and proteomic analysis, have not yet translated to clinical practice. This narrative review aims to identify laboratory biomarkers that have shown significant diagnostic and prognostic value for risk stratification in COVID-19 and discuss the possible clinical application of novel analytic strategies, like metabolomics and proteomics. Future research should focus on identifying a limited but essential number of laboratory biomarkers to easily predict prognosis and outcome in severe COVID-19.

## Introduction

Severe acute respiratory syndrome-coronavirus-2 (SARS-CoV-2) causes a wide spectrum of clinical manifestations, from mild respiratory symptoms to pneumonia and, in more severe cases, multiple organ failure (1). The mechanisms underlying multisystem involvement may include an unbalanced immune response that facilitates the progression of coronavirus disease-2019 (COVID-19). This hypothesis has been confirmed by laboratory biomarker alterations, showing greater potential for abnormal immune response, mainly an increase in neutrophil counts and a substantial reduction in lymphocyte counts, thus altering the neutrophil-to-lymphocyte ratio. Such an abnormal immune response is driven by an increased serum concentration of many pro-inflammatory mediators. These include interleukin (IL)-1β, IL-2, IL-6, IL-8, interferon (IFN)-γ-induced protein 10, granulocyte colony-stimulating factor, monocyte chemoattractant protein 1, macrophage inflammatory protein-1α, and tumor necrosis factor-α, among others (2–5). Nevertheless, the inflammatory cytokine storm in patients with COVID-19 is less injurious than that observed in patients with sepsis or acute respiratory distress syndrome (ARDS) but without COVID-19 (6), thus raising questions regarding the mechanisms underlying multiorgan involvement in COVID-19.

Several biomarkers other than cytokines have been found altered in COVID-19, and are associated with diagnosis, prognosis and outcomes (7). Some of these biomarkers can be easily used to predict disease severity, hospitalization, intensive care unit (ICU) admission, and mortality, while others, like metabolomic and proteomic analysis, are still of purely investigational concern and difficult to translate into clinical practice, despite their prognostic potential (8–10).

The aim of this narrative review is to identify laboratory biomarkers that have shown significant diagnostic and prognostic value for risk stratification in COVID-19 and to discuss the possible clinical application of novel analytic strategies, such as metabolomics and proteomics.

## Potential for Multiorgan Involvement in COVID-19

**Figure 1:**
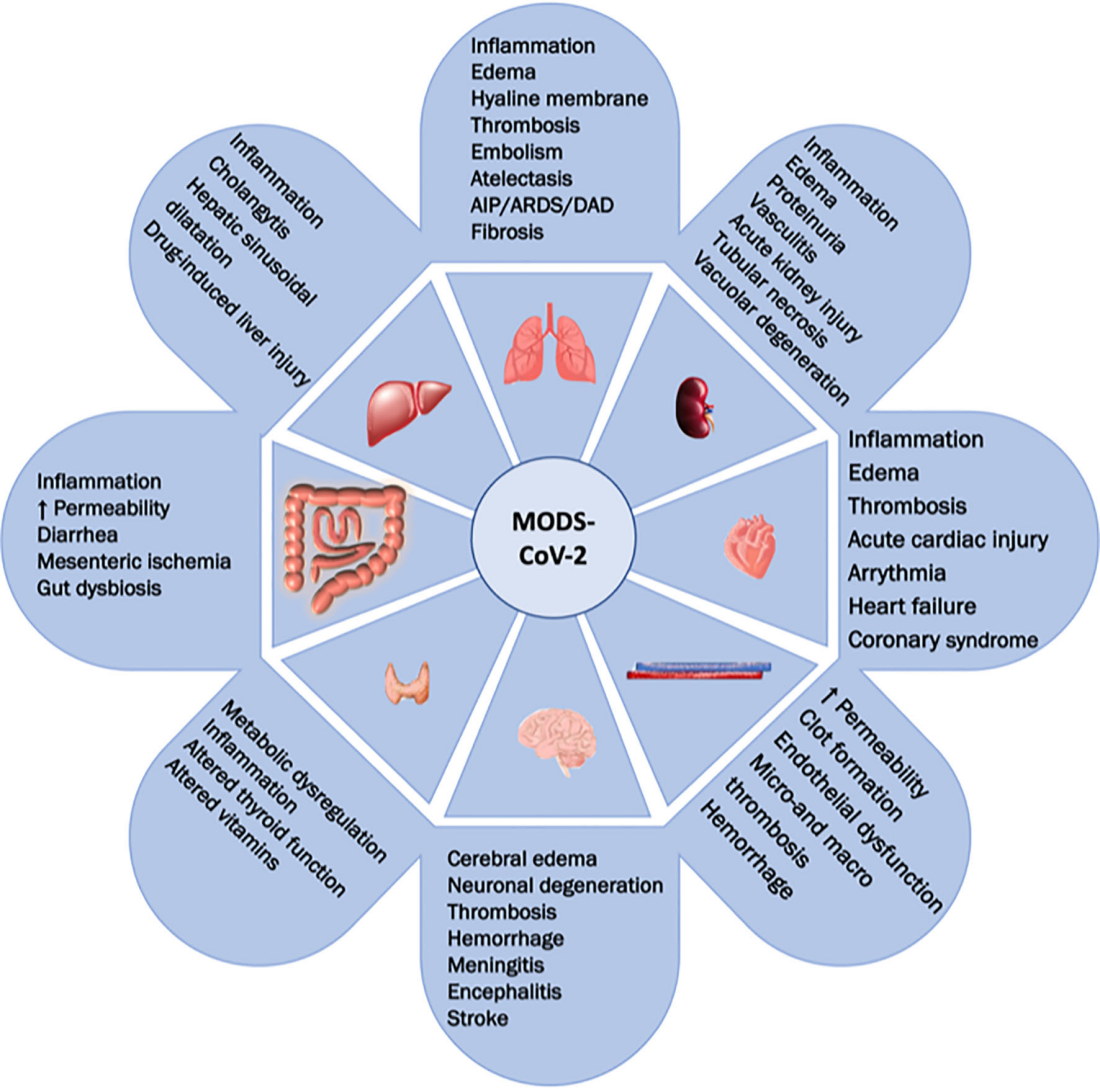
COVID-19 multiple organ dysfunction. This figure shows the potential for multiorgan involvement in COVID-19. Respiratory (AIP, acute interstitial pneumonia; ARDS, acute respiratory distress syndrome; DAD, diffuse alveolar damage), renal, cardiovascular, coagulative/hemostatic, liver, gastrointestinal, metabolic/endocrine, and cerebral functions and systems, as well as their possible alterations, are presented.

SARS-CoV-2 is an enveloped, single-stranded ribonucleic acid (ssRNA) virus. The SARS-CoV-2 genome is composed of two polypeptides encoded between two open-reading frames that are processed by viral proteases to produce nonstructural proteins (11). These proteins are involved in viral replication and suppression of host innate immune defense. On the other hand, structural proteins of SARS-CoV-2 include the spike (S), envelope (E), and nucleocapsid (N) protein, as well as the membrane (M) glycoprotein. The S protein is a transmembrane glycoprotein that is located on the viral surface and cleaved by host-cell proteases. After anchoring the S protein, SARS-CoV-2 enters host cells *via* angiotensin receptor-2 (ACE2), thus activating transmembrane serine protease 2 (TMPRSS2), cathepsin B and L. The E protein is a glycoprotein involved in virion maturation and pathogenesis, while the M protein is involved in viral assembly and delineates the shape of the viral envelope; finally, the N protein binds directly to viral RNA (11). The pathogenic mechanisms of SARS-CoV-2 include 1) direct epithelial damage, 2) dysregulated immune response, 3) ACE2 dysregulation and downregulation of the renin-angiotensin- aldosterone system (RAAS), 4) direct endothelial damage, and, possibly, 5) tissue fibrosis (11). Hence, patients with severe COVID-19 are at high risk of multiple organ involvement and, ultimately, death. Indeed, the virus has been identified in multiple tissues, including endothelial, liver, kidney, pulmonary, and neuronal cells, suggesting direct invasion as possible pathological mechanism underlying systemic effects (1). Therefore, laboratory biomarkers of organ damage play a key role in the diagnosis, prediction, and prognosis of patients at high risk of multiorgan involvement, and their use should be implemented in clinical practice (1). **Table 1** summarizes the most investigated biomarkers in COVID-19, while **Figure 1** depicts possible multiorgan involvement in COVID-19. In the following section, we will describe individual organ systems and how they can be affected by severe COVID-19, associated laboratory and clinical biomarkers of damage, severity, and outcome, and their potential utility for patient management.

**Table 1:** Laboratory biomarkers in COVID-19.

	Biomarkers	Clinical significance
**Pulmonary function**	NSE	Dyspnea
	LDH, AST	Mortality at admission, longer IMV
	Surfactant protein-D, angiopoietin-2, TREM-1, TREM-2	Severity
	Thiol, ferritin, LDH	ARDS development
	Platelet count, neutrophils/lymphocyte ratio, CRP, D-dimer, ferritin	Survival at extubation
	Kynurenine, *p*-cresol sulphate	Longer IMV
	Metabolomic/proteomic: PPAR, D-arginine, D-ornithine, TRP, alpha linoleic	Fibrosis
**Inflammation and infection**	PCT	Severity, mortality
	Neutrophil count	Clinical outcome, mortality
	Neutrophil/lymphocyte ratio	Severity, mortality
	Lymphocyte count, CD3+, 4+, 8+, 25+, 127-, NK cells	Severity, mortality
**Cardiovascular function**	NPs, troponins	CV disease, inflammation, mortality
	MR-proADM	Survival
	CK-MB, myoglobin, D-dimer, BNP, NT-proBNP, neutrophil/lymphocyte ratio	Prognosis
**Coagulation and hemostasis**	D-dimer	Mortality
	Plasma fibrinogen	Hyperinflammation, severity
	sVCAM-1, vWF, thrombomodulin, sTNFRI, HS, C5b9, PAI-1, alpha-2 antiplasmin	Severity
	vWF, ADAMTS13	Mortality
	Endothelial dysfunction	Severity of pulmonary impairment
**Metabolic system**	HDL cholesterol	Risk of hospitalization
	LDL cholesterol	Inflammation
	Vitamin A	ARDS development, mortality
	Metabolomic/proteomic: cAMP	Mortality
	Thyroid hormones	Severity, mortality
**Neurological manifestations**	GFAP, NfL, tau, S100B, NSE, inflammatory markers	Inflammation, severity
	D-dimer, LDH, ESR, CRP, lymphocytes, PCT, creatinine	Occurrence of ischemic stroke
**Kidney and liver function**	Urine 11-dehydro-thromboxane B2, 8-hydroxy-2’-deoxyguanosine, L-FABP	Hospitalization
	N-acetyl-β-D-glucosaminidase, β2-microglobulin, α1-microglobulin, L-FABP	Hyperinflammation
	PCT, arterial saturation of oxygen, blood urea nitrogen	Acute kidney injury
	Creatinine	Acute kidney injury, mortality
	Urine blood, urine weight	Mortality
	Albumin, direct albumin, neutrophils, lymphocytes, mean corpuscular hemoglobin	Severity

ADAMTS, a disintegrin and metalloproteinase with thrombospondin motifs, ARDS, acute respiratory distress syndrome, AST, aspartate aminotransferase, BNP, brain natriuretic peptide, cAMP, adenosine cyclic monophosphate, CD, cluster differentiation, CK-MB, creatine kinase, CRP, C-reactive protein, CV, cardiovascular, ESR, erythrocyte sedimentation rate, GFAP, glial fibrillary acidic protein, HDL, high density lipoproteins, HS, heparan sulfate, IMV, invasive mechanical ventilation, L-FABP, liver-type fatty acid binding protein, LDH, lactate dehydrogenase, LDL, low density lipoproteins, MR-proADM, mid-regional pro-adrenomedullin, NfL, neurofilament light polypeptide, NK, natural killer, NPs, natriuretic peptides, NSE, neuron specific enolase, NT-proBNP, N-terminal pro-hormone, PAI, plasminogen activator inhibitor, PCT, procalcitonin, PPAR, peroxisome proliferator-activated receptors, sTNFRI soluble tumor necrosis factor receptor I, sVCAM-1, vascular cells adhesion molecule-1, TREM, triggering receptor expressed on myeloid cells, TRP, transient receptor potential channel, vWF, von Willebrand.

## Diagnostic and Prognostic Value of Biomarkers

**Figure 2:**
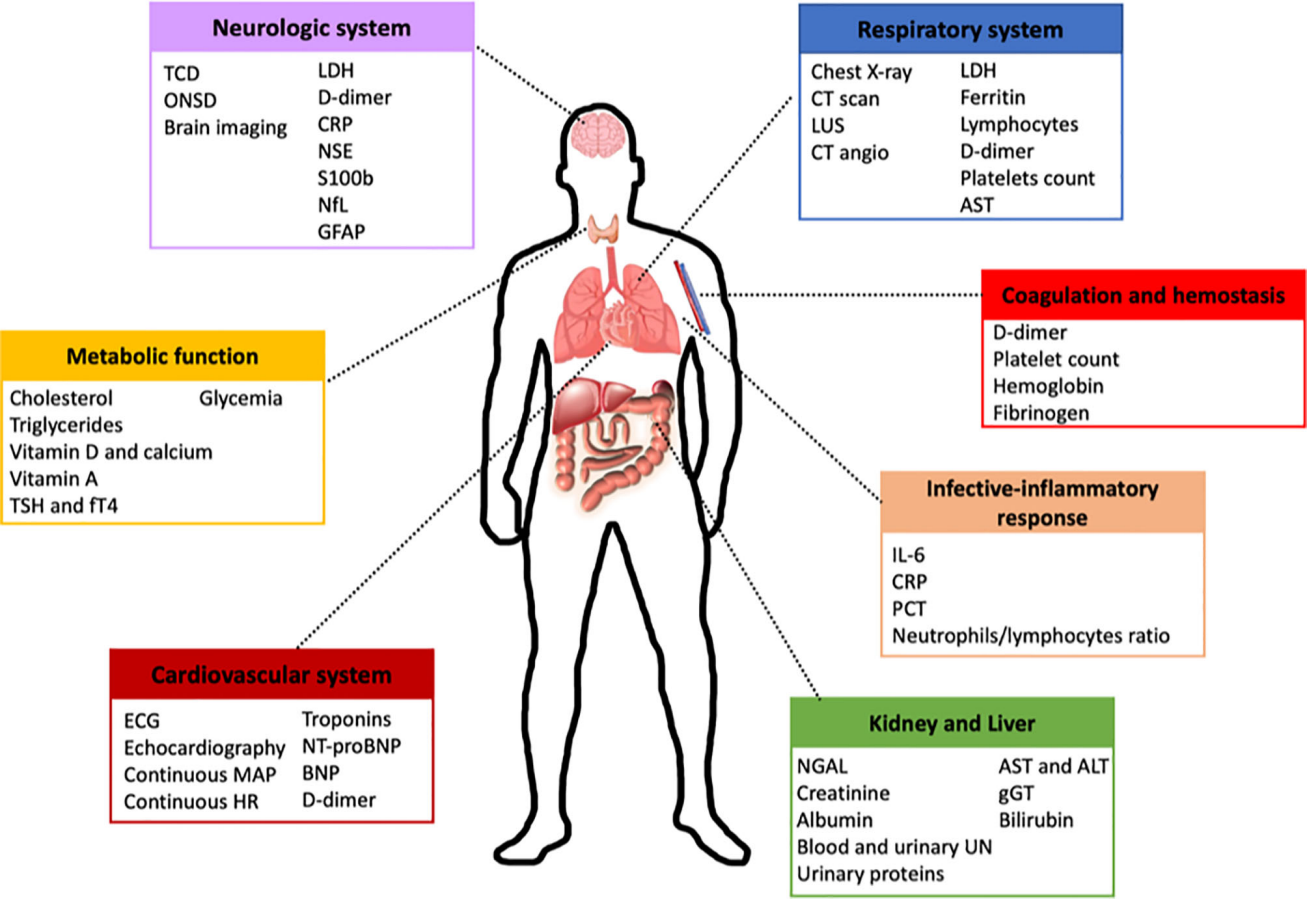
Proposed algorithm for the management of patients with COVID-19 at ICU admission. This figure shows a potential algorithm for initial patient management at ICU admission, including the most useful biomarkers to be used in the COVID-19 critical care setting. Neurological system: sequential transcranial doppler (TCD) and/or optic nerve sheath diameter (ONSD) in sedated patients for whom conventional neurological evaluation is impossible. Cardiovascular system: electrocardiogram and echocardiography, as well as continuous monitoring of mean arterial pressure (MAP) and heart rate (HR), are suggested on ICU admission. Respiratory system: computed tomography (CT) scan is the gold standard; if not feasible, chest X-ray, CT angiography, and/or lung ultrasound should be performed. Lactate dehydrogenase (LDH), C-reactive protein (CRP), neuron specific enolase (NSE), neurofilament light polypeptide (NfL), glial fibrillary acidic protein (GFAP), thyrotropic stimulating hormone (TSH), NGAL, aspartate transaminase (AST), alanine aminotransferase (ALT), gamma-glutamyl transferase (γGT), interleukin-6 (IL-6). BNP, brain natriuretic peptide; UN, urea nitrogen; NT-proBNP, N-terminal pro-hormone.

Biomarkers reflecting multiple organ involvement and/or pharmacological effects have been widely examined in critically ill patients. Some of these biomarkers are also used to monitor dysfunction in distinct organs at the same time, due to their redundancy or non-specificity. However, the most appropriate biomarkers to be studied in critically ill patients with COVID-19 have yet to be defined. **Figure 2** depicts a proposed algorithm for critical care management which includes the investigation of biomarkers in severe COVID-19 patients at ICU admission.

### Respiratory System

The lungs are usually the organs affected primarily by SARS-CoV-2, due to their large and highly vascularized surface area (11). The pathogenesis of COVID-19 in the lung includes an initial phase of local inflammation, endothelial cell damage, and antifibrinolytic activation in the upper and lower respiratory tracts, followed by repair mechanisms that can elicit the restoration of normal pulmonary architecture. Inflammation is followed by platelet recruitment with degranulation, clot formation, altered vessel permeability, and accumulation of leukocytes in the injury site, leading to the recruitment of other inflammatory cells with the involvement of specific cytokines (i.e., IL-4, IL-13, transforming growth factor-β) that are also responsible for pro-fibrotic activity (12).

SARS-CoV-2 lung infection causes a wide variety of clinical manifestations and symptoms, from asymptomatic, mild, and moderate disease to severe COVID-19. Severe and critical illness accounts for up to 14% and 5% of cases, respectively, with the ARDS occurring in 10-20% of patients; multiorgan failure and death may supervene (13, 14). Various phenotypes have been identified by computed tomography (CT) (15, 16), including phenotype L or 1, which is characterized by low compliance, altered ventilation and perfusion, and shunting with focal hypo/hyper-perfused ground-glass opacities; and phenotype H or 2, which is identified by an inhomogeneous distribution of atelectasis with a patchy ARDS-like pattern (17, 18). Progressive evolution of COVID-19 (19) may lead to phenotype F, caused by mechanical stretch of lung epithelial cells and pathological fibro-proliferation and remodeling of the extracellular matrix, with increased expression of pro-fibrotic markers, as is mainly typical of severe forms of lung disease (20).

Although not specific to pulmonary disease, several biomarkers of different stages of lung involvement in COVID-19 have been identified and have been associated with pulmonary and systemic hyperinflammation and fibrotic damage (12). In the early disease course, neuron-specific enolase (NSE) can be used to differentiate patients who are going to develop dyspnea (21). On admission, higher lymphocyte and platelet counts and lower ferritin, D-dimer, lactate dehydrogenase (LDH), and aspartate transaminase (AST) have all been associated with lower risk of mortality in COVID-19 patients who ultimately required intubation and mechanical ventilation (22). Surfactant protein-D, angiopoietin-2, triggering receptor expressed on myeloid cell (TREM)-1, and TREM-2 levels were found to be higher in mild/moderate and severe/critical COVID-19 pneumonia than in asymptomatic and uncomplicated cases. Moreover, these biomarkers correlated well with clinical severity (23, 24). In severe COVID-19 cases, total thiol, ferritin, and LDH were identified as prognostic biomarkers for ARDS development (25). At extubation, COVID-19 survivors had higher platelet counts and neutrophil-to-lymphocyte ratios and lower C-reactive protein (CRP), D-dimer, ferritin, LDH, and AST (22).

### Infection and Systemic Inflammatory Response

Following SARS-CoV-2 invasion of the host cells, the virus replicates at the infection site, thus triggering activation of the innate and adaptive immune responses (26). Neutrophils are rapidly recruited to infection foci, while innate cells recognize the virus and secrete multiple cytokines. Antigen-presenting cells recognize viral antigens which are carried to the local lymph nodes, while activating the T-helper cell response, which is also responsible for stimulating B cells to secrete antibodies (27). The systemic immune-inflammatory response is activated; if left unchecked, this may progress to multiorgan illness (28).

Patients with severe COVID-19 are highly susceptible to superimposed bacterial, fungal, and viral infections, including ventilator-associated pneumonia and bloodstream infection, among others (29, 30). As for systemic biomarkers of infection, procalcitonin is a predictor of disease severity (31), and can be useful to guide antimicrobial stewardship (32, 33). Another study found an association between procalcitonin and mortality in COVID-19 patients more than 75 years old (34). Neutrophil count was also predictive of clinical outcome in hospitalized COVID-19 patients (35), while the neutrophil-to-lymphocyte ratio was strongly associated with severity and mortality in COVID-19 (36). Additionally, total lymphocyte count, cluster differentiation (CD)3^+^, CD4^+^, CD8^+^, CD25^+^, CD127^–^ T cells, and natural killer (NK) cells were found to be depressed in severe COVID-19 (37), whereas C-reactive protein, erythrocyte sedimentation rate, and IL-6 – common markers of inflammation – were elevated (38).

### Cardiovascular System

SARS-CoV-2 can directly trigger endothelial dysfunction, causing a status known as COVID-19-associated coagulopathy. After viral entry into the cells, increased vascular permeability and tissue factor expression in subendothelial cells, with activation of platelets and leukocytes, may trigger the coagulation cascade. Endothelial damage and a generalized inflammatory state are drivers of thrombosis, which can contribute to cardiovascular manifestations (39).

Cardiovascular manifestations of COVID-19 are frequently reported (2, 40). Acute heart failure and exacerbation of chronic heart failure are reported in up to 20-30% of hospitalized patients, and carry high mortality rates, especially in patients with severe comorbidities (41–43). Acute coronary syndrome has been reported in a high proportion of patients, probably because of plaque rupture, coronary spasm, or microthrombi triggered by systemic inflammation and cytokine storm (44). In general, the mechanisms underlying cardiovascular manifestations include increased cardiac workload, hypoxemia, hypervolemia, myocardial injury, arrhythmias, myocarditis, stress-induced cardiomyopathy, acute kidney injury, and, as noted above, systemic inflammatory response with the release of several cytokines and chemokines (45). Triggering mechanisms may be attributed to an imbalance between heightened cardiac workload and reduced oxygen supply secondary to systemic conditions, with possible type-2 myocardial infarction (46).

Cardiac biomarkers (47), electrocardiography (ECG), and transthoracic echocardiography (TTE) play a pivotal role in risk stratification and early detection of cardiovascular complications, as well as to guide treatment (48, 49). Recent evidence confirmed that cardiac biomarkers, including natriuretic peptides (NPs) and troponins, may reflect cardiovascular involvement and inflammation in COVID-19, and are strongly associated with poor prognosis and mortality (41, 50–53). In some cases, troponin elevation in COVID-19 has been associated with ECG changes (54), ICU admission, and in-hospital death (55, 56). However, despite the confirmed prognostic impact of troponins, routine testing is still a matter of debate, because of several other variables that have been associated with outcome and prognosis (48). Additionally, pre-existing cardiac disease and/or acute stress injury may justify mild elevations in cardiac troponins, while myocarditis, Takotsubo syndrome, type 2 myocardial infarction triggered by severe respiratory failure, systemic hypoxemia, or shock are mostly associated with more marked increase in troponins (44, 57, 58). Other cardiac and non-cardiac biomarkers are common findings in COVID-19-associated cardiovascular disease, including creatine kinase (CK)-MB, myoglobin, D-dimer, brain natriuretic peptide (BNP) and its N-terminal pro-hormone (NT-proBNP), and neutrophil-to-lymphocyte ratio (55, 59–61). Myoglobin seems to offer higher prognostic accuracy than other cardiac-specific biomarkers (troponins and CK-MB) in COVID-19 (62). Moreover, mid-regional pro-adrenomedullin (MR-proADM) levels were found to be associated with endothelial dysfunction and mortality in COVID-19, potentially making it an optimal biomarker for the prediction of survival in this patient population (63). Nevertheless, only limited evidence exists so far to define any of these biomarkers as an independent predictor of prognosis in COVID-19 (48, 64).

### Coagulation and Hemostasis

Coagulation derangement is a well-known systemic effect of COVID-19 that can originate from direct or indirect viral impact on the endothelium, or from immunothrombosis (65). COVID-19 can cause alterations in the coagulation cascade, with imbalance of the regulatory mechanisms of coagulation and fibrinolysis, altered platelet function, and a hyperinflammatory response (11, 65). In this context, D-dimer has been identified among the first altered coagulation biomarkers in COVID-19, and is predictive of mortality on admission (66). Similarly, plasma fibrinogen appears to be associated with hyperinflammation and disease severity in COVID-19 (67). A coagulopathy signature diagnostic of COVID-19 has been identified, including elevated levels of soluble vascular cell adhesion molecule (sVCAM)-1 (68), von Willebrand Factor (vWF), thrombomodulin, soluble tumor necrosis factor (TNF) receptor I (sTNFRI), heparan sulfate, C5b9 complement, plasminogen activator inhibitor (PAI)-1, and alpha-2 antiplasmin, among others. Some of these markers, such as sVCAM-1, vWF, sTNFRI, and heparan sulfate, were also associated with disease severity (69). Fibrinogen, thrombin peak, vWF, and ADAMTS13 at admission and elevated vWF : Ag to ADAMTS13 activity ratio were associated with severity and higher risk of death (70, 71). Endothelial dysfunction seems to be persistent after resolution of COVID-19, and directly associated with the severity of pulmonary impairment (72).

### Metabolic Function

Sphingolipid metabolism regulates the inflammation and immune response through the conversion of sphingosine to sphingosine 1-phosphate, increasing the release of lymphocytes into the blood, with subsequent systemic inflammation and release of cytokines and chemokines in COVID-19 (73). Like lipid metabolism, fat-soluble vitamins such as vitamin D have been implicated in suppressing the cytokine storm and enhancing the immune response (74). Investigating lipid metabolism and its biomarkers could thus be of diagnostic and prognostic value in COVID-19.

Metabolic comorbidities including obesity, diabetes, cardiovascular, and hypertension have been associated with poor prognosis in COVID-19 (75). A certain degree of metabolic dysregulation has been found in COVID-19, possibly due to immune-triggered inflammation and hypercoagulability, as well as microbial changes in host physiology (10, 76). Indeed, COVID-19 patients with lower levels of high-density lipoprotein (HDL) cholesterol are more susceptible to hospitalization, while low-density lipoprotein (LDL) cholesterol was associated with higher inflammation (77). Critically ill patients with COVID-19 showed significantly lower levels of vitamin A than non-critical ones, and this was associated with higher inflammation (78). Vitamin A levels below 0.2 mg/L were significantly associated with the developments of ARDS and higher mortality (78). Vitamin D, a well-known regulator of phosphate and calcium metabolism with immunomodulatory functions, seems to not influence mortality or hospital length of stay in COVID-19 (79, 80). Finally, thyroid hormones showed marked association with disease severity and mortality, suggesting the importance of early assessment of thyroid function – and, when necessary, initiation of treatment – in hospitalized COVID-19 patients (81).

### Neurologic Involvement

Pathogenetic mechanisms of SARS-CoV-2 neurologic manifestations include possible spreading of the virus across the blood-brain barrier *via* leukocyte migration or sluggish movement of blood within the microcirculation, thus binding to endothelial cells. Cells which may present ACE2 receptors, including neurons, astrocytes, and oligodendrocytes, can all be affected directly by viral entry and activate the local immune response. As a consequence of neuronal involvement, several biomarkers of neuroinflammation and damage can be detected (82).

Although COVID-19 rarely affects the brain as a primary manifestation, neurological complications are common in this patient population (82–84). Patients with neurological complications, compared to those without, may experience longer hospital stays, and the duration of mechanical ventilation can be associated with the risk of developing new neurological complications (84, 85). CT and magnetic resonance imaging (MRI) are considered the gold standard for detecting cerebral derangements, although the use of methods which involve exposure to ionizing radiation in non-primarily brain-injured patients can only be justified in case of high suspicion of neurological complications (86). The use of multimodal neuromonitoring has received increasing attention as a means of identifying patients at higher risk of brain derangement because of its low cost, speed, safety, and ready availability. However, the use of neuromonitoring tools is still mainly limited to specific settings (i.e., ICU) and patient populations (i.e., those with primary brain injury) (84).

Other than imaging, blood biomarkers can detect brain damage and predict prognosis efficiently. Blood biomarkers for the study of brain derangements include glial fibrillary acidic protein (GFAP), neurofilament light polypeptide (NfL), tau, S100B calcium binding protein, NSE, and inflammatory markers. Increased GFAP staining has been found in postmortem analysis of brain tissue from patients with COVID-19 (87), and NfL was significantly associated with COVID-19 status (88). Another study reported that GFAP was increased in both moderate and severe COVID-19 cases, whereas serum NfL was increased only in severe cases compared to controls (89). However, another study reported that serum NfL, although elevated across patients hospitalized with COVID-19, was not associated with neurological manifestations. Additionally, the usual close correlation between cerebrospinal fluid and serum NfL was not found, suggesting serum NfL elevation in the non-neurological patients may reflect peripheral nerve damage in response to severe illness (90). In COVID-19 patients with altered NfL and GFAP, values of these markers had normalized in all individuals at 6-month follow-up, suggesting that post-COVID-19 neurological sequelae may be not accompanied by ongoing brain injury (91). Inflammatory and coagulatory markers like D-dimer, LDH, erythrocyte sedimentation rate (ESR), and CRP were independently associated with the occurrence of ischemic stroke in COVID-19 (92, 93), while higher age, diabetes mellitus, and hypertension were found not to be significant predictors of stroke in this population, despite being known predictors of non-COVID-19 stroke (93). Levels of lymphocytes, procalcitonin, and creatinine were higher in COVID-19 stroke patients (94). S100B was higher in patients with mild and severe COVID-19 than in healthy controls, and may be a marker of disease severity (95). Antiphospholipid antibodies (i.e., anti-phosphatidylserine/prothrombin) were higher in COVID-19 patients, particularly those with neurological manifestations, than in controls. In contrast, anticardiolipin antibodies were not associated with neurologic involvement in COVID-19 (96).

### Kidney and Liver

COVID-19 may cause kidney and liver injury by either direct infection of cells, *via* host immune clearance and immune tolerance disorders, endothelium-associated vasculitis, thrombus formation, metabolism and glucose disorder, or tissue hypoxia. As a consequence, biomarkers of endothelial, renal, hepatic, vascular, or hypoxic damage can help in the detection of new organ involvement and assist in determining prognosis (97).

As part of multiorgan involvement in COVID-19, kidney function might be altered directly by viral invasion or may occur secondary to multiple organ failure due to systemic inflammation or aggressive therapies (98). Around 25% of patients hospitalized with COVID-19 were reported to develop acute kidney injury, including low molecular weight proteinuria, Fanconi syndrome, and tubular injury (98). Moreover, regional inflammation, endothelial injury, and microthrombi have been identified as major causative factors of renal pathology in COVID-19. This is also sustained by the fact that anti-inflammatory drugs, such as steroids, play a key role in limiting renal disease progression (98). Classic diagnostic biomarkers of kidney damage include creatinine, neutrophil gelatinase-associated lipocalin (NGAL), cystatin C, kidney injury molecule-1 (KIM-1), blood and urinary urea nitrogen, and urinary proteins (99, 100).

Novel urinary biomarkers have been proposed in COVID-19, including urine 11-dehydro-thromboxane B2, 8-hydroxy-2′-deoxyguanosine, and liver-type fatty acid binding protein (L-FABP) levels, all of which were higher in this patient cohort at the time of hospitalization (101). N-acetyl-β-D-glucosaminidase, β2-microglobulin, α1-microglobulin, and L-FABP, which are all markers of tubular injury, were significantly associated with inflammation, as were IL-6 levels (102). Indeed, another observational study confirmed the association between pro-inflammatory cytokines, urinary cytokines, and urinary kidney injury markers (103). Procalcitonin was associated with acute kidney injury in COVID-19, and a score including simple and easily accessible variables such as procalcitonin, arterial saturation of oxygen, and blood urea nitrogen was shown to be predictive of acute kidney injury (104).

Altered serum creatinine levels with decreased kidney function at admission and up to 24 hours thereafter were significantly associated with acute kidney injury and in-hospital mortality (105). Additionally, urine blood >0.03 mg/dL and urine specific gravity >1.026 were associated with acute kidney injury, ICU admission, and higher mortality (106).

Abnormal liver and hepatobiliary function have been also identified in COVID-19 (107). A systematic review and meta-analysis showed a cumulative prevalence of liver disease of 24% in COVID-19, with possible alterations in albuminemia, liver enzymes, and total bilirubin (108). Recent findings showed that some liver and renal biomarkers, including albumin, direct bilirubin, neutrophil and lymphocyte counts, and mean corpuscular hemoglobin, are associated with risk of developing severe COVID-19 (107). Moreover, the presence of pre-existing liver fibrosis with silent liver injury significantly influenced mortality in COVID-19 (109).

## Future Perspectives: Metabolomic and Proteomic Biomarkers and Machine Learning Models

Given the significant immune dysregulation of COVID-19 patients, the interplay between metabolism and immunity may play a pivotal role in the disease course (110). Additionally, oxygen deprivation may affect homeostasis in tissues and organs such as the lung, brain, kidney, and liver. The modulation of oxygen homeostasis and response to hypoxia is mainly mediated by glycolysis and the lactate cycle. This has increased research interest in proteomic and metabolomic methods to investigate pathways linked to energy production and amino acid metabolism in patients with SARS-CoV-2 infections (110). Metabolomic analyses in COVID-19 patients with and without pulmonary fibrosis revealed that pathways including the peroxisome proliferator-activated receptor (PPAR), D-arginine and D-ornithine metabolism, inflammatory tryptophan metabolic pathway (TRP), and alpha-linolenic acid metabolism were significantly increased in fibrotic lungs, thus suggesting that PPAR signaling is one of the main pathways involved in the formation and development of lung fibrosis in COVID-19 (9). A proteomic and metabolomic analysis identified hypoxanthine and betaine as predictors of ICU stay, and early ICU admission, elevated creatinine, and D-dimer were found to be associated with these pathways (8). Longer duration of invasive mechanical ventilation was associated with the kynurenine and *p*-cresol sulfate pathways (8). Several markers of metabolic function identified *via* metabolomic analysis were associated with in-hospital mortality, including cyclic adenosine monophosphate (cAMP), which plays a role in SARS-CoV2 endocytosis in the initial phase of the disease (10). Another major signature of the serum metabolome in COVID-19 was lactic acid, as well as spermidine and spermine. Many other metabolites were commonly increased, including glutamate, aspartate, phenylalanine, β-alanine, ornithine, arachidonic acid, choline, and xanthine (110). Recent machine learning models have been developed to support decision making and risk stratification in COVID-19. Most predictive models rely on demographic and clinical variables. However, biomarkers have recently shown good correlation with severity of disease and mortality in COVID-19 modeling (111). One example was a large study of 2,895 consecutive patients with COVID-19 in whom three biomarkers measured at admission were found to reflect pathobiological axes of myocardial injury, altered coagulation, and inflammation. The machine learning model concluded that patients with low levels of these biomarkers were at lower risk of critical disease and in-hospital mortality (112). In conclusion, the alterations found in the serum metabolome of patients with COVID-19 may reflect a more complex systemic derangement affecting carbon and nitrogen liver metabolism, but further research is needed to completely understand the impact of these alterations on routine clinical practice. Machine learning models can be promising in risk stratification in COVID-19. However, further investigations are needed to develop mathematical models that can help clinicians select the right parameters and interpret results.

## Conclusions

Laboratory biomarkers have shown significant diagnostic and prognostic value for risk stratification in COVID-19. Furthermore, novel analytic strategies including metabolomics and proteomics offer interesting insights for early detection of patients at higher risk of severe disease and death. However, their limited availability restricts their widespread clinical use. Further investigations are warranted to identify a core set of laboratory biomarkers which can be used in daily clinical practice to easily predict prognosis and outcome in hospitalized patients with severe COVID-19.

## Author Contributions

DB and ML-P: review, design, writing, editing. HC-F-N and PP: editing. PR: review, design, editing, senior contribution. All authors contributed to the article and approved the submitted version.

## Funding

This work was supported by the Brazilian Council for Scientific and Technological Development (COVID-19-CNPq; 401700/2020-8 and 403485/2020-7); Rio de Janeiro State Research Foundation (COVID-19-FAPERJ; E-26/210.181/2020); and Funding Authority for Studies and Projects (01200008.00), Brazil.

## Conflict of Interest

The authors declare that the research was conducted in the absence of any commercial or financial relationships that could be construed as a potential conflict of interest.

## Publisher’s Note

All claims expressed in this article are solely those of the authors and do not necessarily represent those of their affiliated organizations, or those of the publisher, the editors and the reviewers. Any product that may be evaluated in this article, or claim that may be made by its manufacturer, is not guaranteed or endorsed by the publisher.

## Abbreviations

ACE2, angiotensin receptor 2; ARDS, acute respiratory distress syndrome; AST, aspartate transaminase; BNP, brain natriuretic peptide; cAMP, cyclic adenosine monophosphate; CD, cluster differentiation; CK, creatine kinase; COVID-19, coronavirus disease 2019; CRP, C-reactive protein; CT, computed tomography; ECG, electrocardiography; ESR, erythrocyte sedimentation rate; GFAP, glial fibrillary acidic protein; HDL, high-density lipoprotein; ICU, intensive care unit; IL, interleukin; KIM, kidney injury molecule; L-FABP, liver-type fatty acid binding protein; LDH, lactate dehydrogenase; LDL, low-density lipoprotein; MR-proADM, mid-regional pro-adrenomedullin; MRI, magnetic resonance imaging; NfL, neurofilament light polypeptide; NGAL, neutrophil gelatinase-associated lipocalin; NK, natural killer; NP, natriuretic peptides; NSE, neuron-specific enolase; NT-proBNP, N-terminal pro-hormone BNP; PPAR, peroxisome proliferator-activated receptor; RASS, renin-angiotensin- aldosterone system; SARS-CoV-2, severe acute respiratory syndrome-coronavirus-2; ssRNA, single-stranded ribonucleic acid; sTNFRI, soluble TNF receptor I; sVCAM, soluble vascular cell adhesion molecule; TMPRSS2, transmembrane serine protease 2; TNF, tumor necrosis factor; TREM, triggering receptor expressed on myeloid cell; TRP, tryptophan metabolic pathway; TTE, transthoracic electrocardiography; vWF, von Willebrand Factor.
